# Pre‐Encoded IFN‐I Sensitivity Exacerbates Memory T Cell Senescence in Solid Tumors

**DOI:** 10.1002/advs.202504474

**Published:** 2025-10-05

**Authors:** Andrew Nguyen, Scott R Walsh, Li Deng, Lan Chen, Yonghong Wan

**Affiliations:** ^1^ Department of Medicine, Centre for Discovery in Cancer Research McMaster University Hamilton Ontario L8N 3Z5 Canada

**Keywords:** cancer immunotherapy, oncolytic virus, T cell senescence, tumor microenvironment, type I interferon

## Abstract

Solid tumors often suppress antitumor immune responses by promoting various dysfunctional CD8+ T cell states, which limit the effectiveness of T‐cell‐based immunotherapy. However, the mechanisms that promote these states have not been fully characterized. It is demonstrated that spontaneous priming responses during tumor growth can produce memory T cell reservoirs that are conducive to poor proliferative responsiveness during boosting vaccination. Surprisingly, when type I interferon (IFN‐I) signaling is impeded, boosting vaccination can elicit robust proliferative responses from tumor‐primed memory T cells and promote tumor control. This is observed in multiple tumor types and target antigens. In contrast to conventional memory T cells, tumor‐primed memory T cells are unique in their pre‐encoded responsiveness to IFN‐I and show enrichment of pathways pertaining to DNA repair and cell cycle arrest. Tumor‐primed memory T cells up‐regulate p21 expression and blockade of either p21 or IFN‐I can alleviate this effect to improve their proliferative capacity during boosting vaccination. Characterization of tumor‐primed memory T cells revealed transcriptional and phenotypic features of cellular senescence, where higher senescence severity correlated with higher responsiveness to IFNα/β receptor blockade. Overall, IFN‐I hyperresponsiveness may be a unique feature of senescent tumor‐primed memory T cells that can exacerbate their dysfunction during cancer vaccination.

## Graphical abstract

Type I interferon (IFN‐I) signaling promotes p21‐dependent cell cycle arrest in senescent tumor‐specific memory T cells, resulting in poor proliferative responses and solid tumor regression during cancer vaccination. Conversely, IFNα/β receptor blockade reinvigorates T cell proliferation to regress solid tumors and is more effective with increasing senescence severity. Overall, IFN‐I hyperresponsiveness is identified as a pre‐encoded feature of severely senescent tumor‐specific memory T cells.

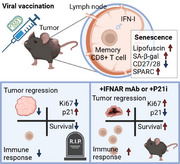

## 1 Introduction

Type I interferons (IFN‐I) are pleiotropic cytokines best known for their role in limiting viral replication, but can also regulate T cell responses in a multimodal capacity. During antigen‐specific naïve T cell priming, they are pivotal for up‐regulating co‐stimulatory molecules on antigen presenting cells and for providing pro‐proliferative and/or survival signals in T cells^[^ ^1^ ^]^ Defects in IFN‐I signaling have been associated with compromised antiviral responses and tumor therapy.^[^ ^2^, ^3^, ^4^ ^]^ As a result, IFN‐I are often a major component of the cancer vaccine inflammatory profile and have been traditionally associated with enhanced vaccination efficacy.^[^ ^5^ ^]^ Indeed, many early cancer vaccines were intentionally designed to engage IFN‐I pathways using toll‐like receptor agonists (e.g., Poly I:C, LPS)^[^ ^6^, ^7^, ^8^, ^9^ ^]^ or viruses encoding IFN‐I directly (e.g., VSV‐IFNα/β).^[^ ^10^, ^11^, ^12^ ^]^ In contrast to naïve T cell priming, it is unclear whether IFN‐I is beneficial for the secondary expansion of memory T cells. For instance, while detriments in secondary T cell proliferation were not observed after LCMV exposure in IFNα/β receptor (IFNAR) knock‐out mice, there was observable impact on viral control.^[^ ^13^ ^]^ However, we have recently reported that IFNAR blockade did not affect the magnitude or quality of secondary T cell responses generated from adoptive memory T cell transfer (ACT) followed by oncolytic viral vaccination (OVV) in solid tumor‐bearing mice. In presence or absence of IFNAR signaling, we observed potent regression of tumors, suggesting that IFN‐I was dispensable.^[^ ^14^ ^]^

Modern cancer immunotherapeutic approaches often rely on the secondary expansion of endogenous tumor antigen‐specific memory T cells. In the clinic, endogenous tumor antigen‐specific memory T cell reservoirs were identified in cancer patients and shown to undergo proliferative responses during therapeutic cancer vaccination or immune checkpoint blockade (ICB) therapy.^[^ ^15^, ^16^, ^17^ ^]^ Since they have the capability to infiltrate and reject solid tumors in an antigen‐agnostic manner, endogenous tumor antigen‐experienced T cells, which we term “tumor‐primed memory T cells” (TP‐T_M_), are invaluable as stimulation targets for mounting effective antitumor responses and for preventing evasion by antigen‐negative tumor cell variants.^[^ ^18^, ^19^, ^20^ ^]^ However, TP‐T_M_ can display considerable phenotypic differences compared to more conventionally formed memory T cells. In contrast to memory T cells formed by acute infection with a virus (acute virus‐primed memory T cells (VP‐T_M_) or by in vitro peptide stimulation of naïve T cells for ACT (cultured T_M_), TP‐T_M_ are formed spontaneously during tumor growth and are exposed to disseminated immunosuppressive factors from the solid tumor microenvironment (TME).^[^ ^21^ ^]^ This can result in dysfunctional T cell states that can blunt the secondary expansion and function of TP‐T_M_‐derived antitumor responses.^[^ ^22^ ^]^ Indeed, TP‐T_M_ were found to have molecular/functional similarities to memory‐like cells in chronic viral infections which suggests a state of “cellular exhaustion”.^[^ ^23^, ^24^, ^25^, ^26^ ^]^ However, while immunotherapy approaches have been shown to be potent enough to provide sufficient proliferative signaling to reinvigorate “exhausted” TP‐T_M_‐derived responses, therapeutic resistance to cancer vaccination/ICB has also been extensively reported.^[^ ^27^, ^28^ ^]^ This variable responsiveness of TP‐T_M_ to immunotherapy may suggest that TP‐T_M_ dysfunction is heterogeneous^[^ ^29^ ^]^ and that disproportionate enrichment of dysfunctional T cell states distinct from cellular exhaustion may contribute to treatment failure. As such, identifying these states and their characteristics will be key to the design of more effective cancer immunotherapeutic approaches.

Recently, it was determined that treatment failure and diminished survival time during ICB therapy was strongly associated with T cell hyperresponsiveness to IFN‐I.^[^ ^30^ ^]^ If this is a TP‐T_M_‐intrinsic feature, this could indicate that certain tumors can propagate unique dysfunctional T cell states such that IFN‐I abrogates immunotherapy‐driven T cell responses. Blocking IFN‐I signaling may therefore rescue the therapeutic efficacy of ICB and other immunotherapy approaches that engage the secondary expansion of TP‐T_M_. In this study, we reconcile the role of IFN‐I in cancer immunotherapy by examining how it differentially affects the proliferation of conventional T_M_ compared to TP‐T_M_ during vaccination‐driven secondary T cell responses. By blocking IFN‐I signaling, we were able to augment the proliferation and therapeutic efficacy of TP‐T_M_ to enhance the survival of solid tumor‐bearing mice. We demonstrate that TP‐T_M_ express the transcriptional and phenotypic characteristics of cellular senescence and suggest that sensitivity to IFNAR blockade treatment correlates with senescence severity. Overall, IFN‐I may be a detriment for cancer vaccines by reinforcing replicative senescence during secondary expansion of TP‐T_M_.

## 2 Results

### 2.1 Type I IFN Selectively Obstructs Endogenous T Cell Expansion while IFNAR Blockade Augments Antitumor Responses and Therapeutic Efficacy

We have repeatedly demonstrated that combination immunotherapy consisting of ACT and OVV can regress solid tumors through the secondary expansion of transferred memory T cells.^[^ ^19^, ^31^, ^32^ ^]^ At the same time, endogenous tumor‐specific T cells may also undergo expansion as a result of epitope spreading and are pivotal for preventing tumor recurrence.^[^ ^18^ ^]^ While we have demonstrated that IFN‐I signaling may be dispensable for ACT‐driven secondary T cell responses,^[^ ^14^ ^]^ it is unclear whether endogenous T cell responses share a similar property.

**Figure 1:**
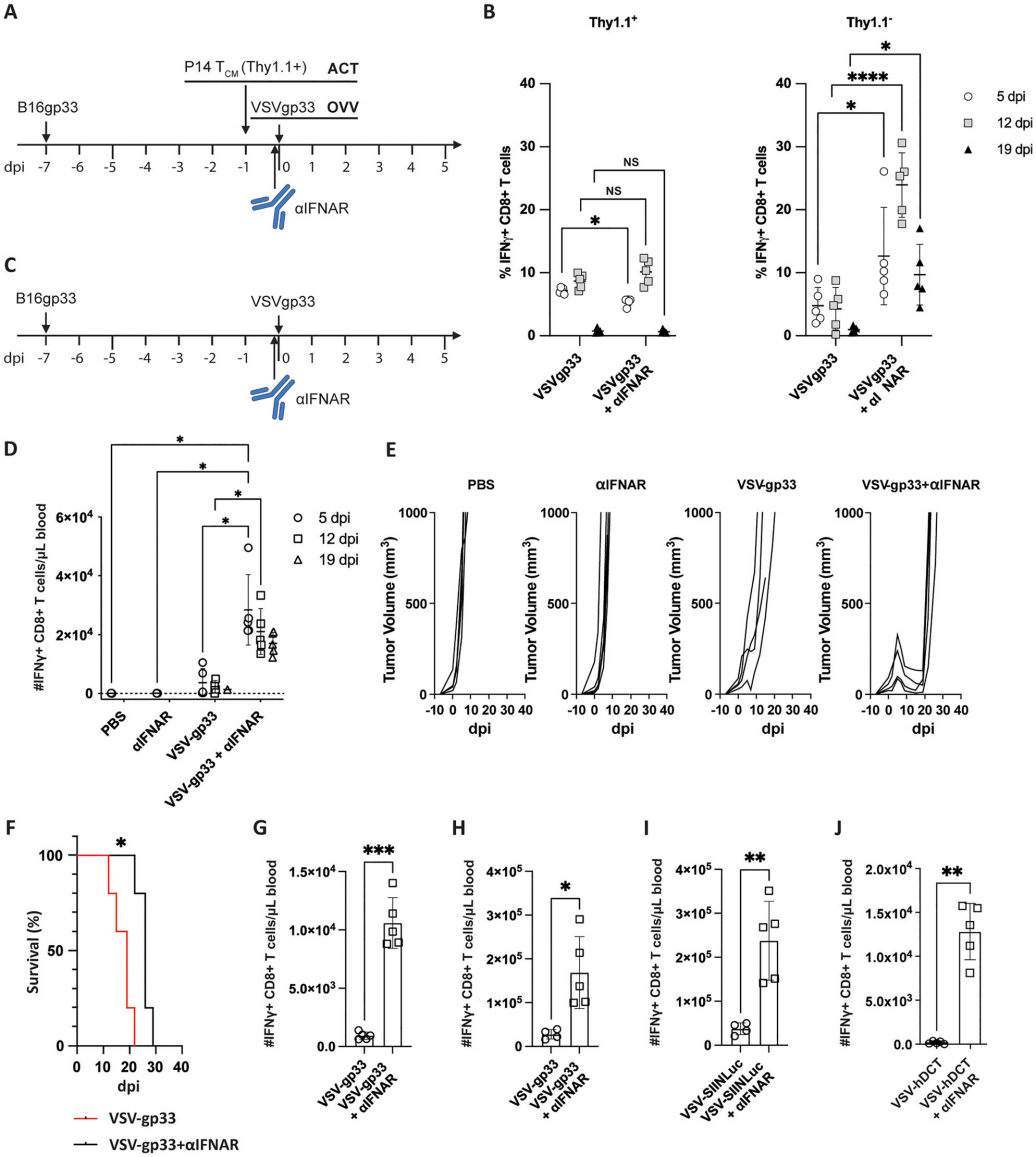
IFNAR blockade augments responses derived from endogenous T cells in multiple tumor‐bearing models. A,C) Thy1.1‐ C57BL/6 mice were implanted i.d. with B16‐gp33 tumor cells and allowed to grow for 7 days before being infected with VSV‐gp33 i.v. ± αIFNAR mAb i.p. (A) One day prior to infection, mice were infused i.v. with T_CM_‐cultured Thy1.1+ P14 T cells. B,D) At 5, 12, and 19 dpi, peripheral blood was drawn to assess gp33‐specific T cell frequency/number by *ex vivo* peptide stimulation and IFNγ staining. (D) Endogenous (Thy1.1‐)/exogenous (Thy1.1+) CD8+ T cell responses were separated based on Thy1.1 staining. E) Tumor growth was monitored and tumor volume (mm^3^) was determined by length x width x height F) Survival curves. (G‐I) VSV ± IFNAR blockade treatment in different tumor models including G) LLCgp33, H) MCA205gp33, I) M05(SIINFEKL), and (H) B16F10 (DCT/TRP2) tumors. At 5 dpi, antigen‐specific T cell numbers were quantified as previously described. Data are representative of at least two experiments with n = 5 mice per group. Statistical significance was defined as an FDR < 0.05 (*), FDR < 0.001 (**), FDR < 0.005(***), FDR < 0.0001 (****). Error bars represent the SEM, and statistics were based on an unpaired t‐test, one‐way ANOVA, or log‐rank test. dpi = days post‐infection, ACT = adoptive memory T cell transfer, OVV = oncolytic viral vaccination, NS = no significance.

B16‐gp33 tumors (B16F10 melanoma cell line expressing gp33, a lymphocytic choriomeningitis virus (LCMV) glycoprotein‐derived peptide) were treated with ACT + OVV (adoptive transfer of in vitro differentiated P14 memory T cells (T_CM_) that recognize the gp33 epitope followed by vaccination with vesicular stomatitis virus (VSV) expressing gp33 (VSV‐gp33)) in the presence or absence of transient anti‐IFNAR antibody exposure (**Figure** **1**a). Through congenic marker staining, we observed that transferred T cells (Thy1.1+) showed no change in response with IFNAR blockade while endogenous T cells (Thy1.1‐) showed dramatically enhanced responses with IFNAR blockade (Figure 1b). This suggests that endogenous tumor‐specific T cell expansion may be selectively impaired by IFN‐I signaling.

Given their apparent sensitivity to IFN‐I signaling, pre‐existing endogenous tumor‐specific T cells may be able to have a primary therapeutic role during immunotherapy in the context of IFNAR blockade. To evaluate this possibility, we repeated the experiment in the absence of ACT (Figure 1c). PBS and IFNAR antibody alone treatment controls showed minimal responses against gp33 at five days post‐infection (dpi) (Figure 1d) and minimal tumor control (Figure 1e). VSV‐gp33 treatment induced a small gp33‐specific response, peaking at five dpi, (Figure 1d), which corresponded with a minor controlling effect on tumor growth between three and 10 dpi (Figure 1e). However, responses were short‐lived and diminished to a nominal level by 12 dpi accompanied by robust outgrowth of the tumor (Figure 1d,e). Interestingly, combination of IFNAR blockade with VSV‐gp33 vaccination substantially augmented gp33‐specific T cell responses compared to VSV‐gp33 alone (>7‐fold increase), mediated significant tumor regression, and prolonged survival (Figure 1d–f).

We then extended our findings into different tumor models and target antigens. LLC (Lewis lung carcinoma) and MCA205 (fibrosarcoma) cells were engineered to express the gp33 peptide (LLC‐gp33 and MCA205‐gp33, respectively). After tumor challenge and vaccination, we observed that concurrent IFNAR blockade significantly increased the magnitude of gp33‐specific endogenous T cell responses compared to virus alone (Figure 1g,h). MO5 (B16F10 melanoma cells engineered to express ovalbumin (OVA) protein) tumor‐bearing mice were treated with VSV expressing SIINFEKL (VSV‐SIIN), an OVA‐derived immunodominant epitope, or VSV expressing DCT/TRP2, a B16F10‐intrinsic melanocyte differentiation antigen. With IFNAR blockade, we once again observed an increase in tumor‐specific endogenous T cell responses during vaccination (Figure 1i,j). Taken together, these results confirm that endogenous tumor‐specific T cells have potent antitumor and proliferative capacity during vaccination, but are hampered by IFN‐I signaling.

### 2.2 Tumor‐Primed Memory T Cells Display Differential Responsiveness to IFN‐I Signaling Compared to Conventional Memory T Cells During Secondary Expansion

**Figure 2:**
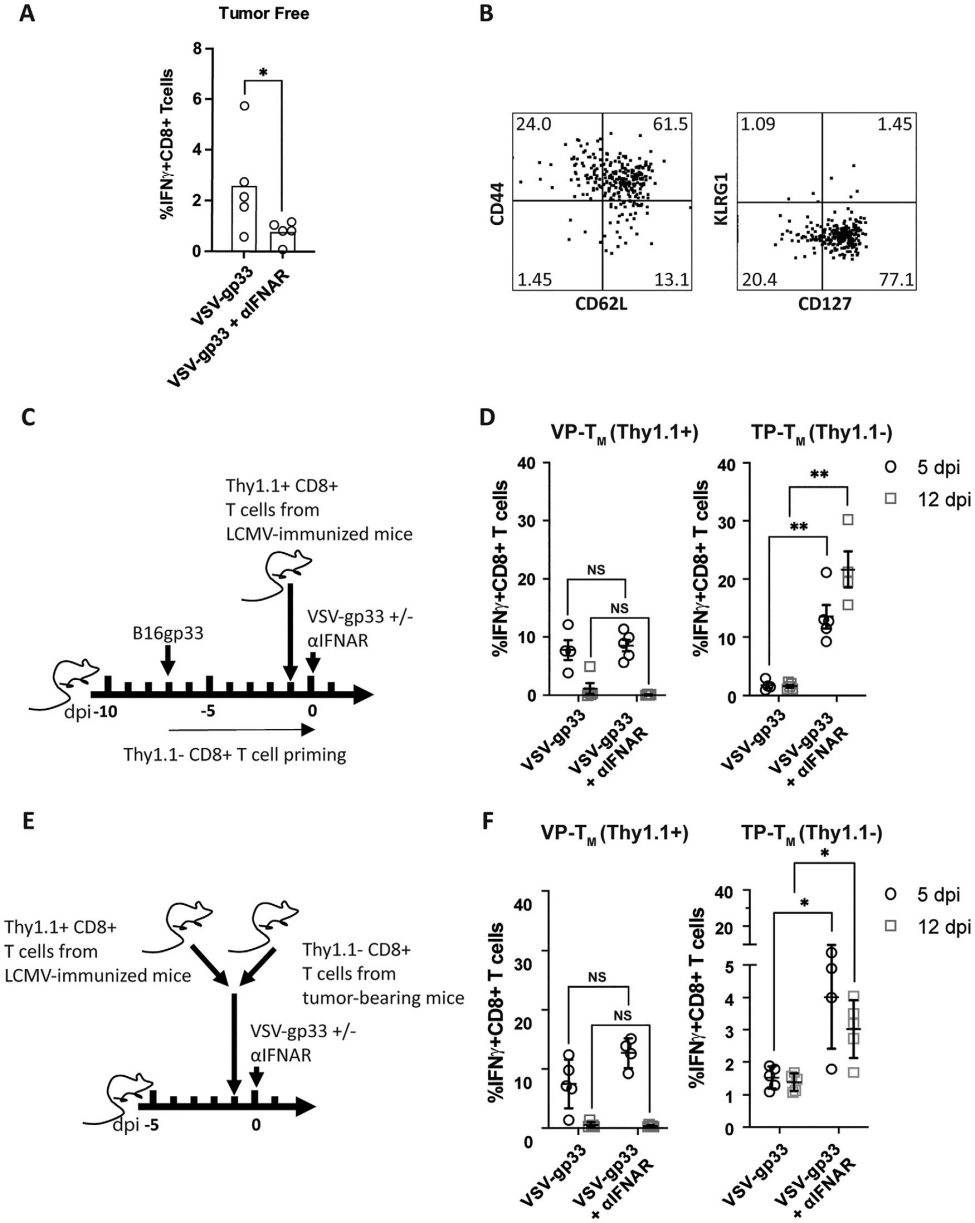
Secondary expansion of endogenous tumor‐primed memory T cells is selectively enhanced by IFNAR blockade. A) Tumor‐free mice were treated with VSV‐gp33 treatment ± IFNAR blockade. At 5 dpi, peripheral blood was drawn and gp33‐specific T cell frequency was assessed by ex vivo peptide stimulation and IFNγ staining. B) C57BL/6 mice were challenged i.d. with B16‐gp33 tumors for 7 days prior to gp33‐specific CD8+ T cell isolation from the tumor‐draining lymph nodes. Isolated cells were stained with gp33 tetramer and an antibody panel of T cell differentiation markers. C) Thy1.1‐ C57BL/6 mice were implanted i.d. with B16‐gp33 tumor cells and allowed to grow for 7 days before being infected with VSV‐gp33 i.v. ± αIFNAR mAb i.p. One day prior to infection, mice were infused i.v. with Thy1.1+ CD8+ T cells enriched from LCMV‐immunized mice. D,F) At 5 or 12 dpi, endogenous Thy1.1‐ (TP‐T_M_)/exogenous Thy1.1+ (VP‐T_M_) CD8+ T cell responses were separated based on Thy1.1 staining and quantified as previously described. E,F) In tumor‐free mice, mice were infused i.v. with Thy1.1+ CD8+ T cells enriched from LCMV‐immunized mice and Thy1.1‐ CD8+ T cells enriched from 7d C57BL/6 tumor‐bearing mice. After one day, they were infected with VSV‐gp33 i.v. ± αIFNAR mAb. Data are representative of at least two experiments with n = 4–5 mice per group. Statistical significance was defined as an FDR < 0.05 (*) or FDR < 0.001 (**). Error bars represent the SEM, and statistics were based on an unpaired t‐test or one‐way ANOVA. dpi = days post‐infection, VP‐T_M_ = virus‐primed memory T cells, TP‐T_M_ = tumor‐primed memory T cells, NS = no significance.

While endogenous T cell responses may be invaluable for antitumor immunity, it is unclear why they behave differently in response to IFN‐I and vaccination compared to cultured T_M_ for ACT. As previously described, if endogenous T cells responses are the result of vaccine‐driven naïve T cell priming then theoretically IFN‐I signaling should enhance the response rather than hamper it. To confirm this, we vaccinated tumor‐free mice with VSV‐gp33 and observed that IFNAR blockade reduced gp33‐specific T cell responses (**Figure** **2**a), suggesting that endogenous T cell responses during ACT + OVV treatment of tumor‐bearing mice were not priming responses.

If endogenous T cell responses are representative of secondary responses, then the tumor must play an integral role to facilitate naïve T cell priming, likely through the cross‐presentation of tumor‐derived antigens during growth. This will lead to the formation of a tumor antigen‐experienced T cell reservoir that can be re‐expanded through vaccination. Using a gp33 tetramer, we identified pre‐existing tumor‐specific endogenous CD8+ T cells from the tumor‐draining lymph nodes of untreated B16‐gp33 tumor‐bearing mice, and identified these cells as having a classical central memory T cell phenotype with high expression of CD44, CD62L, and CD127, and low expression of KLRG1 (Figure 2b). Therefore, in vitro‐cultured T_M_ and endogenous tumor‐primed memory T cells (TP‐T_M_) may both undergo secondary expansion during OVV boosting, but intrinsic differences may affect how they respond to IFN‐I signaling. We hypothesized that IFN‐I being dispensable for secondary T cell responses may be an inherent feature of conventional memory T cells, such as those derived from in vitro culture or acute viral infection. By contrast, disseminated immunosuppressive factors from within a growing TME may influence memory formation to create a unique antigen‐experienced T cell reservoir that poorly responds to vaccination in the presence of IFN‐I. To test that IFN‐I hyperresponsiveness is a unique feature of TP‐T_M_ that is absent from conventional memory T cells, we compared the proliferative behavior of both TP‐T_M_ and VP‐T_M_ (generated via acute infection with LCMV‐Armstrong) during vaccination and IFNAR blockade from within the same host. We transferred VP‐T_M_ from infected Thy1.1+ mice into Thy1.1‐ B16‐gp33 tumor‐bearing mice (wherein TP‐T_M_ are spontaneously formed) (Figure 2c) and used the congenic marker to differentiate TP‐T_M_ and VP‐T_M_ expansion after vaccination. Similar to what we previously observed with ACT + OVV, secondary gp33‐specific VP‐T_M_ responses did not change in magnitude with IFNAR blockade; however, TP‐T_M_ once again showed enhanced gp33‐specific responses in mice that received IFNAR blockade (Figure 2d).

We then wondered whether TP‐T_M_ embodied an encoded T cell state from tumor priming or if the presence of the tumor was required for continued maintenance of this behavior. We repeated the experiment in tumor‐free mice which received an equal co‐transfer of Thy1.1‐ TP‐T_M_ (isolated from the tumor‐draining lymph nodes of B16‐gp33 tumor‐bearing mice) and Thy1.1+ VP‐T_M_ (Figure 2e). Once again, VP‐T_M_ responses showed little change while TP‐T_M_ responses showed a significant increase when IFNAR blockade was combined with VSV vaccination (Figure 2f). Taken together, these results indicated that endogenous T cell responses were derived from pre‐existing tumor‐primed memory T cells that were uniquely encoded for hyperresponsiveness to IFN‐I signaling, which in turn was associated with poor proliferative/therapeutic outcomes during vaccination.

### 2.3 TP‐T_M_ are Characterized by DNA Damage Response‐/Cell Cycle Arrest‐Related Pathway Enrichment Converging upon the p21/CDKN1A Signaling Axis

If TP‐T_M_ demonstrate reduced proliferative and therapeutic capacity during vaccination unless IFNAR blockade is concurrently administered, then in contrast to conventional memory T cells, TP‐T_M_ may exemplify a state of cellular dysfunction. Studies have observed that TP‐T_M_‐like cells can have molecular/functional characteristics of cellular exhaustion;^[^ ^33^, ^34^ ^]^ however, flow cytometry staining using classical T cell exhaustion markers showed no elevation in the expression of Tim‐3, Lag‐3, CD38, and CD39 in TP‐T_M_ compared to VP‐T_M_, though PD‐1 was more highly expressed (Figure S1, Supporting Information). Moreover, IFN‐I hyperresponsiveness is not typically an observed trait of exhausted T cells.^[^ ^30^ ^]^

**Figure 3:**
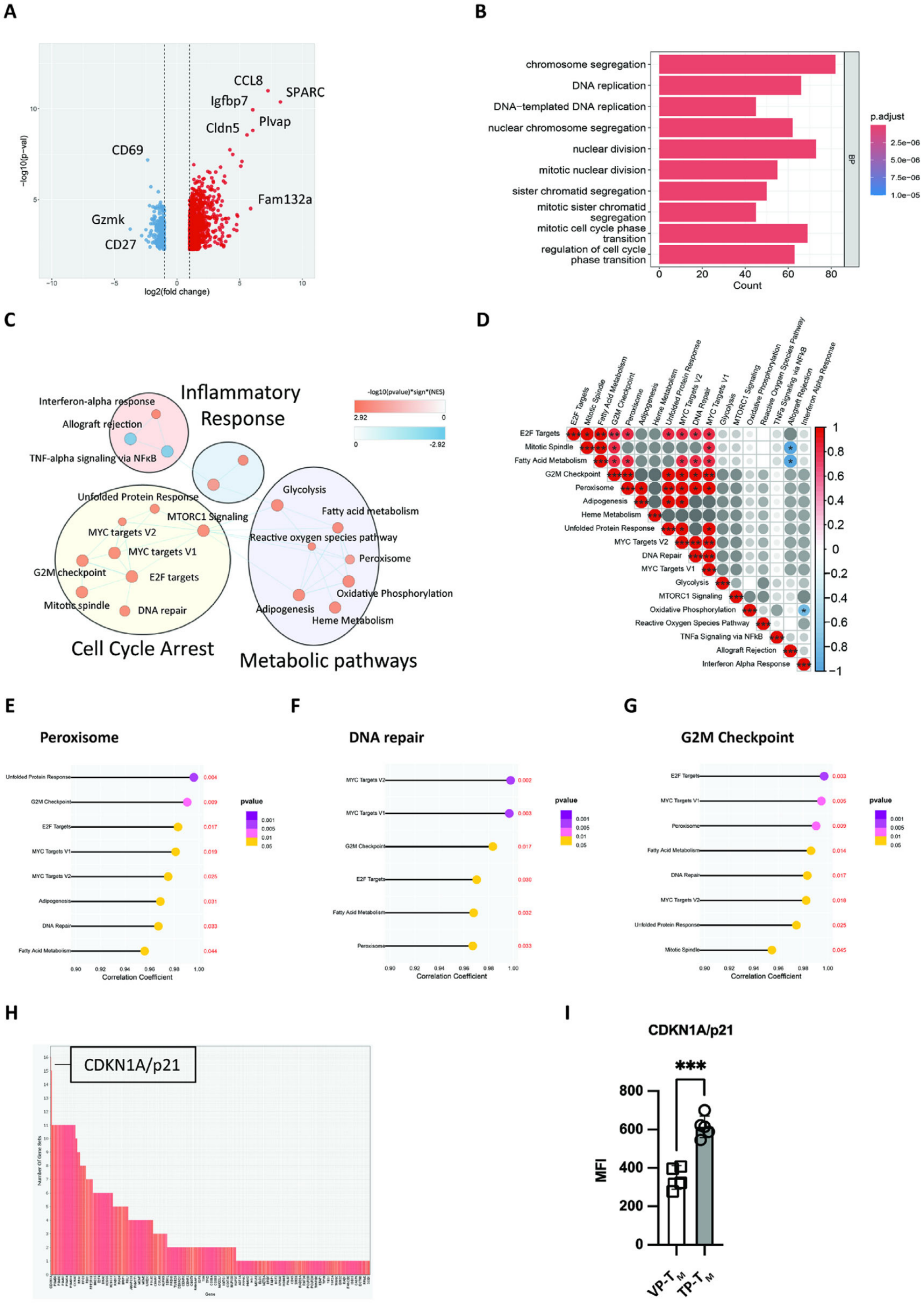
Tumor‐primed memory T cells show enrichment of p21/CDKN1A‐dependent cell cycle arrest‐related pathways. C57BL/6 mice were challenged i.d. with B16‐gp33 tumors in order to generate TP‐T_M_ or acutely infected i.v. with LCMV in order to generate virus‐primed memory VP‐T_M_. Memory T cells were isolated from secondary lymphoid tissue and sorted based on gp33H2DbTet^+^ CD8^+^ staining. RNA was extracted from purified cells for whole transcriptome analysis. A) Volcano plot of differentially expressed genes (>2‐fold change) when comparing TP‐T_M_ to VP‐T_M_. Labels were used to identify top up‐regulated or down‐regulated genes. B) Top gene ontology BP terms that are overrepresented in TP‐T_M_ versus VP‐T_M_. C) Enrichment map with annotation after conducting gene set enrichment analysis (GSEA) comparing TP‐T_M_ to VP‐T_M_ using human ortholog Hallmark gene sets from the Molecular Signatures Database (MSigDB). The color scale reflects the normalized enrichment score where red is up‐regulation and blue is down‐regulation. D) Pathway correlation matrix after conducting single‐sample GSEA with Hallmark gene sets using TP‐T_M_ samples. Colored circles represent statistically significant correlations (FDR < 0.05) and the color scale represents the correlation coefficient where red is positive correlation and blue is negative correlation. E–G) Statistically significant positive correlations of Hallmark pathways against the designated reference pathway. H) Leading edge analysis after conducting GSEA comparing TP‐T_M_ to VP‐T_M_ using MSigDB Curated gene sets (C2) corresponding to DNA damage and cell cycle arrest. I) TP‐TM and VP‐T_M_ were isolated from the tumor draining lymph nodes and flow stained for CDKN1A/p21 expression. Data are representative of at least one experiment with n = 4 mice per group. (I) At n = 5, the statistical significance was defined as an FDR < 0.005 (***). Error bars represent the SEM, and statistics were based on an unpaired t‐test. VP‐T_M_ = virus‐primed memory T cells, TP‐T_M_ = tumor‐primed memory T cells, BP = biological processes, NES = normalized enrichment score, MFI = mean fluorescence intensity.

To characterize dysfunction in TP‐T_M_, using RNA extracted from purified TP‐T_M_ and VP‐T_M_, we conducted whole transcriptome analysis and measured differential gene expression between the two groups. After ranking differentially expressed genes by fold‐change, our analysis revealed that the majority of genes were up‐regulated in TP‐T_M_, including *SPARC* (304‐fold), *CCL8* (153‐fold), *PLVAP* (66‐fold), *IGFBP7* (66‐fold), *FAM132A* (59‐fold), and *CLDN5* (48‐fold) (**Figure** **3**a). By contrast, relatively few genes were down‐regulated, the most apparent being *GZMK* (‐13‐fold), *CD27* (‐7.1‐fold), and *CD69* (‐5.1‐fold). When filtering for genes that showed at least two‐fold change, gene ontology (GO) overrepresentation analysis revealed that the most significant GO terms pertained to the regulation of cell cycle progression (Figure 3b). Gene set enrichment analysis (GSEA) using Hallmark pathway gene sets from the Molecular Signatures Database (MSigDB) confirmed that multiple pathways that were selectively enriched in TP‐T_M_ were indeed cell‐cycle related, specifically cell‐cycle arrest‐related (Figure 3c). This corresponded with the profound up‐regulation of many cellular metabolic pathways. Interestingly, while inflammatory responses were negatively enriched in TP‐T_M_, the “interferon‐alpha response” was positively enriched. This observation aligns with the apparent hypersensitivity of TP‐T_M_ to IFN‐I such that they do not proliferate during boosting vaccination unless IFNAR blocking antibodies are administered. To infer whether the IFN‐I signaling can directly regulate any of the other enriched pathways, we utilized ssGSEA scores of the Hallmark pathways to create a correlation matrix (Figure 3d). However, while “interferon‐alpha response” was negatively correlated with “oxidative phosphorylation”, we did not observe positive correlation with any other pathways, suggesting that IFN‐I likely contributes to non‐proliferation in an indirect manner during boosting vaccination.

We did observe significant correlation in other enriched pathways, which gave some indication why TP‐T_M_ undergo cell cycle arrest. Indeed, positive correlation between “peroxisome”, “DNA repair”, and “G2M checkpoint” pathways (Figure 3e–g) led us to infer that, sequentially, cellular and genotoxic stress in TP‐T_M_ may activate a DNA repair response and promote cell cycle arrest. We repeated the GSEA using MSigDB C2 manually curated pathways that were filtered for cell cycle‐arrest‐related and DNA damage‐related pathways and saw similar positive enrichment in TP‐T_M_ (Figure S2, Supporting Information). Leading‐edge analysis indicated that *CDKN1A* (p21) was the most represented leading‐edge gene (Figure 3h), suggesting that p21 expression may be highly correlated with this pathway phenotype. Indeed, when comparing TP‐T_M_ to VP‐T_M_ by flow cytometry, p21 expression was significantly higher in TP‐T_M_ (Figure 3i).

### 2.4 IFN‐I Signaling Contributes to p21‐Dependent Cell Cycle Arrest while IFNAR Blockade Restores Cellular Proliferation in TP‐T_M_

**Figure 4:**
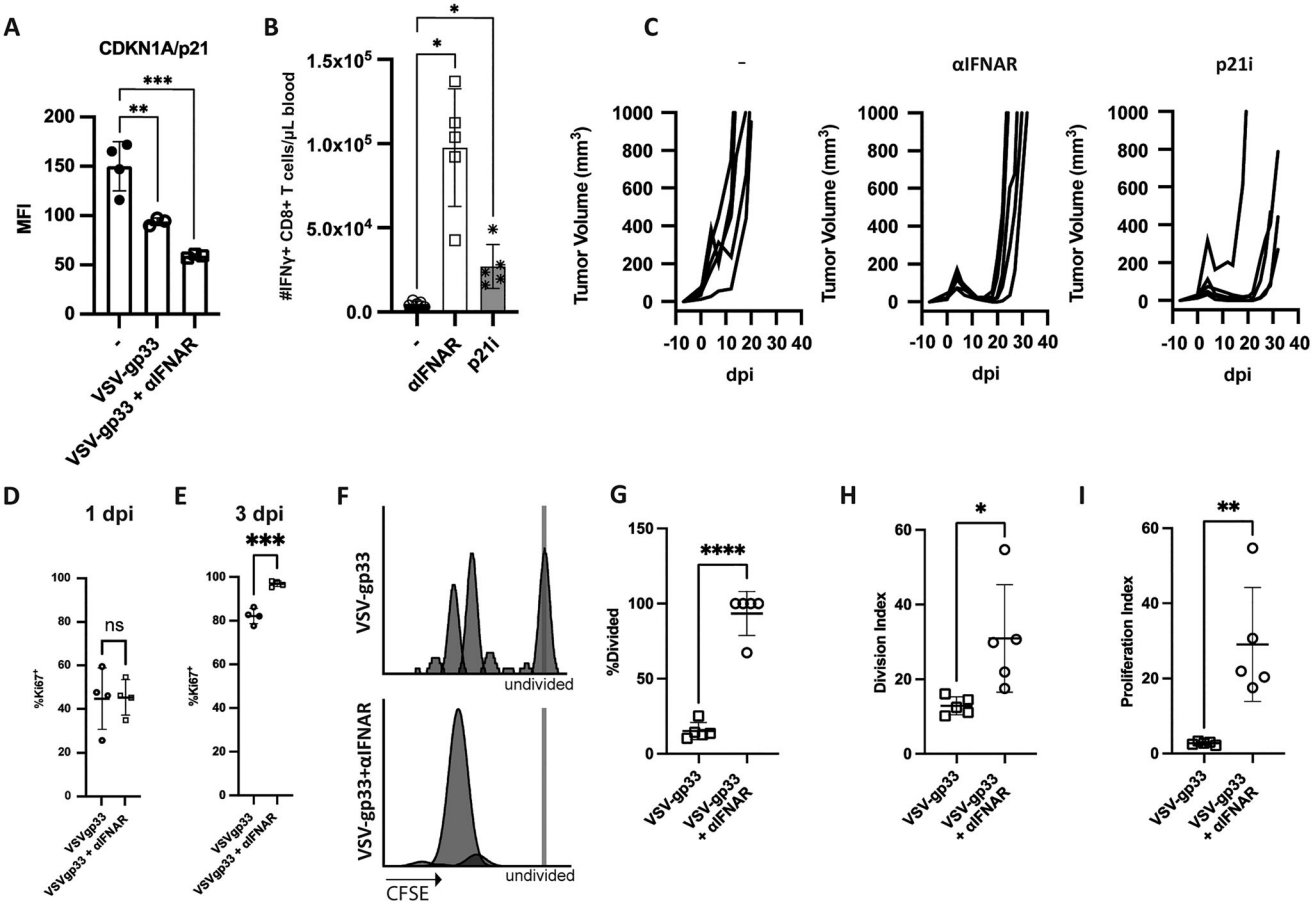
IFNAR blockade down‐regulates CDKN1A/p21 and restores the proliferation of tumor‐primed memory T cells during secondary expansion. A) C57BL/6 mice were implanted i.d. with B16‐gp33 tumor cells and allowed to grow for 7 days before being infected with VSV‐gp33 i.v. ± αIFNAR mAb i.p. At 5 dpi, peripheral blood was drawn and CDKN1A/p21 expression in gp33H2DbTet^+^CD8^+^ cells was assessed by flow cytometry. B) A similar experiment was conducted whereby a selective p21 inhibitor, UC2288, was injected i.p. in place of αIFNAR mAb. At 5 dpi, peripheral blood was drawn and gp33‐specific T cell numbers were assessed by ex vivo peptide stimulation and IFNγ staining. C) Tumor growth was monitored and tumor volume (mm^3^) was determined by length x width x height. D,E) In another similar experiment, at (D) one dpi or (E) three dpi, gp33H2DbTet^+^ CD8^+^ T cells were isolated from tumor draining lymph nodes and stained for Ki67. F–I) C57BL/6 mice were implanted i.d. with B16‐gp33 tumor cells and allowed to grow for 7 days. gp33H2DbTet^+^ CD8^+^ T cells were isolated from tumor draining lymph nodes, labeled with CFSE, and infused i.v. into tumor‐free mice followed by treatment. (F) Representative plot showing the CFSE dilution of labelled TP‐T_M_ from peripheral blood at 5dpi. G–I) Quantitative analysis of CFSE dilution showing the (G) proportion of cells having divided, (H) division index values, and (I) proliferation index. Data are representative of at least two experiments with n = 4–5 mice per group. Statistical significance was defined as an FDR < 0.05 (*), FDR < 0.001 (**), FDR < 0.005(***), FDR < 0.0001 (****). Error bars represent the SEM, and statistics were based on an unpaired t‐test or one‐way ANOVA. dpi = days post‐infection, TP‐T_M_ = tumor‐primed memory T cells, = mean fluorescence intensity, NS = no significance.

We have demonstrated that TP‐T_M_ are hyperresponsive to IFN‐I signaling, which corresponds to pre‐encoded dysfunctional characteristics (represented by higher p21 expression) that manifest during boosting vaccination. The fact that IFNAR blockade improves T cell responses and therapeutic efficacy suggests that IFN‐I signaling could have some overall impact on p21 expression and cell cycle arrest. To determine if IFN‐I signaling regulates p21 expression, we conducted flow cytometry staining in TP‐T_M_ after treating B16p33 tumor‐bearing mice with VSV‐gp33 and IFNAR blocking antibody. We observed that the expression of p21 had decreased with just VSV‐gp33 treatment alone (**Figure** **4**a), which was consistent with our prior finding that vaccination alone could still stimulate an unprotective, low‐magnitude, endogenous T cell response. More importantly, IFNAR blockade greatly reduced the expression of p21 (Figure 4a), confirming that IFN‐I signaling may up‐regulate p21 to potentiate cell cycle arrest and limit the secondary expansion of TP‐T_M_. To show that improved therapeutic efficacy associated with IFNAR blockade is attributed to the inhibition of p21‐dependent cell cycle arrest, we used a selective p21 inhibitor (UC2288) during vaccination and were able to recapitulate the therapeutic effects of IFNAR blockade on both gp33‐specific T cell response (Figure 4b) and tumor regression (Figure 4c), albeit not to the same degree of effectiveness.

If TP‐T_M_ embody an anti‐proliferative state which is largely regulated by IFN‐I signaling, IFNAR blockade may enhance gp33‐specific T cell responses through restoration of cellular proliferation. Indeed, when we isolated gp33‐specific TP‐T_M_ from the tumor‐draining lymph nodes after vaccination, we observed a progressive, relative increase in the proportion of cells that expressed proliferation marker, Ki67, with IFNAR blockade from one dpi to three dpi (Figure 4d,e). Moreover, when isolated TP‐T_M_ from B16‐gp33 tumor‐bearing mice were labeled with proliferation dye, transferred into tumor‐free mice, and treated with VSV‐gp33, there was a greater dilution of proliferation dye with IFNAR blockade (Figure 4f). This corresponded to a higher frequency of cells that had entered division (Figure 4g) and a higher average number of divisions in total (Figure 4h) or amongst divided cells (Figure 4i). Overall, the data indicate that TP‐T_M_ largely fail to divide during vaccination but have renewed proliferative capacity through IFNAR blockade.

### 2.5 The Effectiveness of IFNAR Blockade is Tumor Type‐Dependent

**Figure 5:**
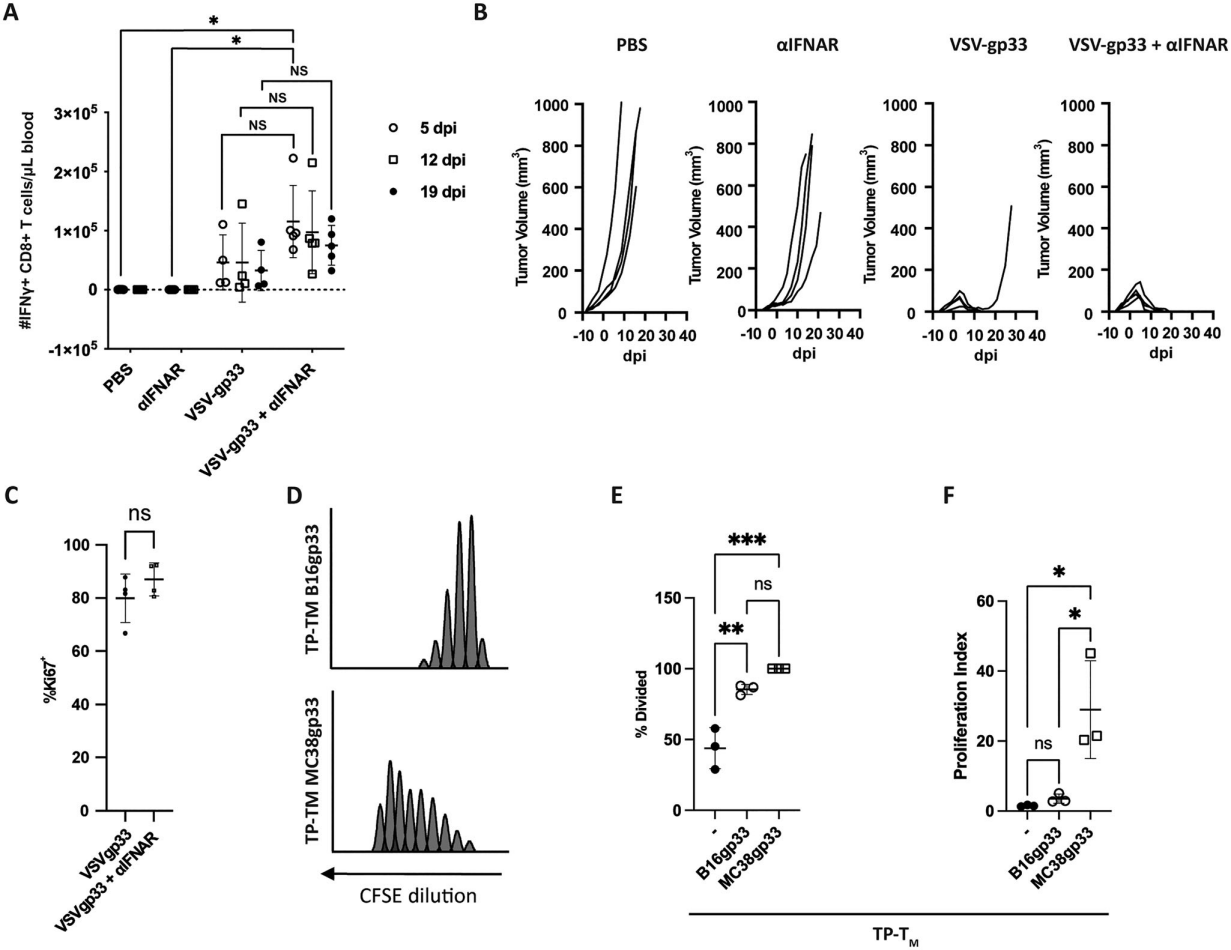
Proliferative benefit of IFNAR blockade during viral vaccination depends on the relative dysfunction of tumor‐primed memory T cells. A) C57BL/6 mice were implanted i.d. with MC38‐gp33 tumor cells and allowed to grow for 7 days before being infected with VSV‐gp33 i.v. ± αIFNAR mAb i.p. At 5, 12, and 19 dpi, peripheral blood was drawn and gp33‐specific T cell frequency was assessed by *ex vivo* peptide stimulation and IFNγ staining B) Tumor growth was monitored and tumor volume (mm^3^) was determined by length x width x height. C) In a similar experiment, gp33H2DbTet^+^ CD8^+^ T cells were isolated from tumor draining lymph nodes at three dpi and stained for Ki67. D–F) C57BL/6 mice were implanted i.d. with B16‐gp33 or MC38‐gp33 tumor cells and allowed to grow for 7 days. gp33H2DbTet^+^ CD8^+^ T cells were isolated from tumor draining lymph nodes, labeled with CFSE, and infused i.v. into tumor‐free mice followed by VSV‐gp33 infection. (D) Representative plot showing the CFSE dilution of labelled TP‐T_M_ from peripheral blood at 5dpi. (E,F) Quantitative analysis of CFSE dilution showing the (E) proportion of cells having divided and (F) proliferation index. Data are representative of at least two experiments with (A–C) n = 4–5 (D‐F) n = 3 mice per group. Statistical significance was defined as an FDR < 0.05 (*), FDR < 0.001 (**), FDR < 0.005(***). Error bars represent the SEM, and statistics were based on an unpaired t‐test or one‐way ANOVA. dpi = days post‐infection, TP‐T_M_ = tumor‐primed memory T cells, NS = no significance.

In our previously described tumor models, we observed that IFNAR blockade consistently improved endogenous T cell responses during vaccination. Further characterization suggested that antigen‐experienced memory T cell reservoirs primed by tumor growth were inherently dysfunctional, uniquely characterized by IFN‐I hyperresponsiveness, and did not proliferate well during secondary expansion; however, this may not always be the case. When we attempted to extend our findings to an MC38‐gp33 tumor model (colon adenocarcinoma), we were surprised to see robust endogenous T cell responses with VSV‐gp33 vaccination alone (**Figure** **5**a) that were sufficient to regress established tumors (Figure 5b). Although there was an increase (albeit statistically insignificant) in gp33‐specific endogenous T cell responses with IFNAR blockade, there was no observable difference in tumor regression (Figure 5a,b). Similarly, there was no significant increase in Ki67 staining (Figure 5c), suggesting that gp33‐specific endogenous T cells in MC38‐gp33 tumors do not respond particularly well to IFNAR blockade. Although vaccination of MC38‐gp33 tumors also promoted the secondary expansion of endogenous TP‐T_M_ (Figure S3a–c, Supporting Information), the quality of TP‐T_M_ may not be uniform across tumor types. We postulated that TP‐T_M_ derived from MC38‐gp33 (TP‐T_M_^MC38^) may differ from TP‐T_M_ derived from B16‐gp33 (TP‐T_M_^B16^) in their embodiment of pre‐encoded dysfunction. Indeed, when isolated TP‐T_M_^MC38^ and TP‐T_M_^B16^ from tumor‐bearing mice were labeled with proliferation dye and stimulated with gp33 peptide *ex vivo*, there was greater dilution in TP‐T_M_^MC38^ (Figure 5d). While the frequency of cells that had entered division was roughly equal across both groups (Figure 5e), TP‐T_M_^MC38^ demonstrated a higher average number of divisions amongst divided cells (Figure 5f). Taken together, intrinsic differences between tumor types may dictate whether TP‐T_M_ will be more or less dysfunctional, which in turn may affect their responsiveness to IFNAR blockade.

### 2.6 IFNAR Blockade‐Sensitive TP‐T_M_ Display Greater Cellular Senescence Severity

**Figure 6:**
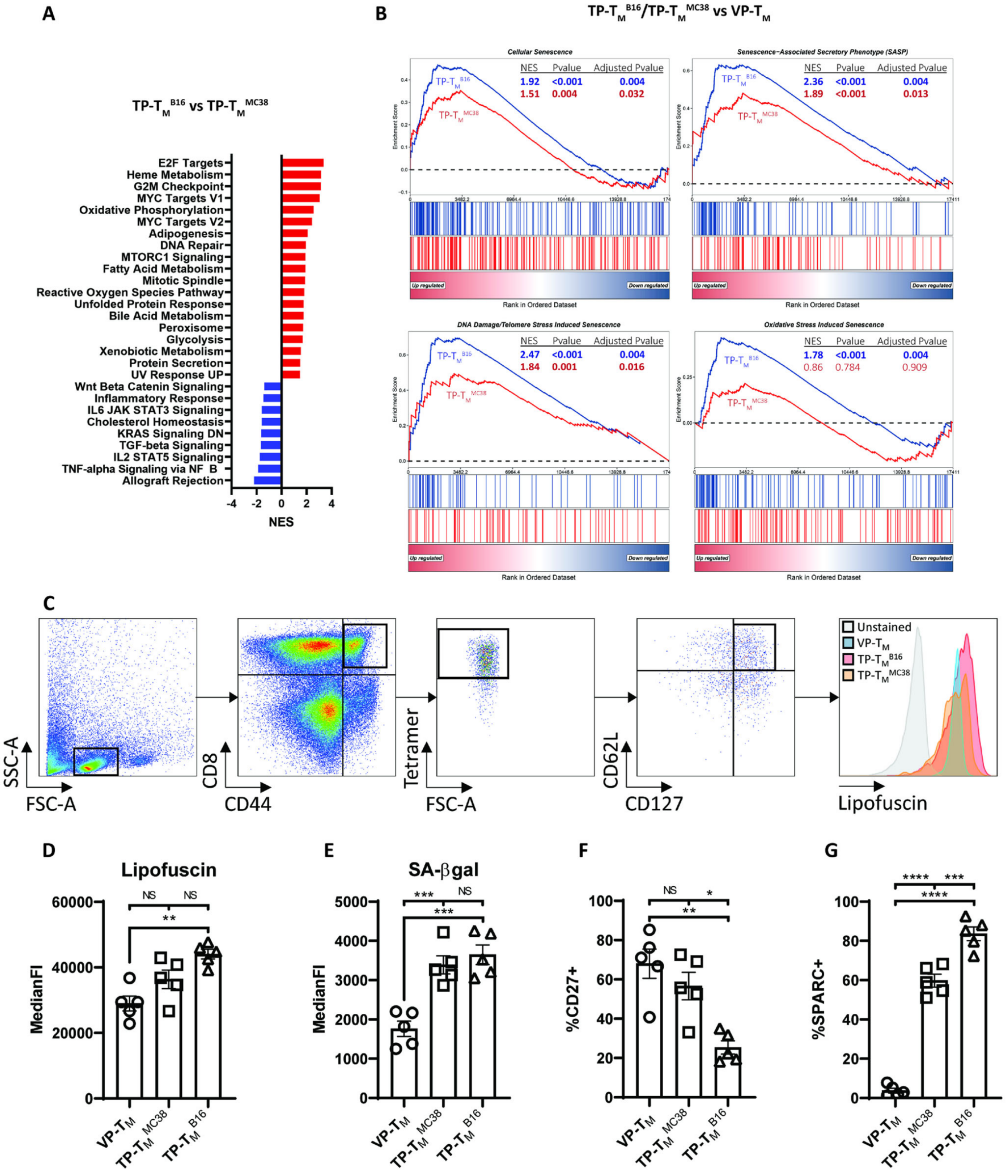
IFNAR blockade sensitivity of tumor‐primed memory T cells correlates with cellular senescence severity. C57BL/6 mice were challenged i.d. with B16‐gp33/MC38‐gp33 tumors in order to generate TP‐T_M_^B16^/ TP‐T_M_^MC38^ or acutely infected i.v. with LCMV in order to generate VP‐T_M_. Memory T cells were isolated from secondary lymphoid tissue and sorted based on gp33H2DbTet^+^ CD8^+^ staining. RNA was extracted from purified cells for whole transcriptome analysis. A) GSEA comparing TP‐T_M_^B16^ to TP‐T_M_^MC38^ using human ortholog MSigDB Hallmark gene sets. Red denotes a positive NES and up‐regulation while blue denotes a negative NES and down‐regulation. B) GSEA comparing TP‐T_M_^B16^ versus VP‐T_M_ to TP‐T_M_^MC38^ versus VP‐T_M_ enrichment using senescence‐related gene sets taken from the Reactome database (Table S1, Supporting Information). Each enrichment plot represents a senescence‐related gene set where at least TP‐T_M_^B16^ or TP‐T_M_^MC38^ have shown up‐regulation relative to VP‐T_M_. C–G) TP‐T_M_^B16^, TP‐T_M_^MC38^, and VP‐T_M_ were generated and isolated as previously described where (C) CD8+ CD44+ gp33H2DbTet+ CD62L+ CD127+ cells were flow stained for known senescence markers including (D) lipofuscin, (E) SA‐β‐gal, (F) CD27, and (G) SPARC. Data are representative of at least (A‐B) one or (C‐G) two experiments with (A‐B) n = 4 (C‐G) n = 5 mice per group. Statistical significance was defined as an FDR < 0.05 (*), FDR < 0.001 (**), FDR < 0.005(***), FDR < 0.0001(****). Error bars represent the SEM, and statistics were based on an unpaired t‐test or one‐way ANOVA. TP‐T_M_^B16^ = B16‐gp33‐derived tumor‐primed memory T cells, TP‐T_M_^MC38^ = MC38‐gp33‐derived tumor‐primed memory T cells, VP‐T_M_ = virus‐primed memory T cells, NES = normalized enrichment score, NS = no significance, medianFI = median fluorescence intensity.

To assess cellular dysfunction in TP‐T_M_^B16^ and TP‐T_M_^MC38^, we used RNA extracted from purified TP‐T_M_^B16/MC38^ (with VP‐T_M_ as a control) to conduct whole transcriptome analysis. GSEA comparing TP‐T_M_^B16^ and TP‐T_M_^MC38^ using MSigDB Hallmark pathway gene sets showed enrichment patterns that were similar to when we previously compared TP‐T_M_^B16^ to VP‐T_M_. Specifically, cell cycle arrest/DNA repair and metabolic pathways were once again up‐regulated in TP‐T_M_^B16^ (**Figure** **6**a), suggesting that relatively, TP‐T_M_^MC38^ did not suffer from the exacerbated p21‐dependent cell cycle arrest that we identified in TP‐T_M_^B16^. By contrast, inflammatory response‐related pathways were up‐regulated in TP‐T_M_^MC38^ (Figure 6a), suggesting that these cells retained their ability to engage in an antitumor immune response and agrees with our prior observation that VSV‐gp33 treatment alone could regress MC38‐gp33 solid tumors. Taken together, TP‐T_M_^MC38^ are similar to VP‐T_M_ in that they do not share the same level of pre‐encoded pathway dysfunction as TP‐T_M_^B16^.

Although we previously identified that TP‐T_M_^B16^ were not exhausted, it was still unclear what dysfunctional cell state was being expressed by these cells. We reasoned that the pathway changes we identified that culminated in p21‐dependent cell cycle arrest could be uniquely tied to a known state of cellular dysfunction. T cell senescence is often characterized by persistent DNA damage responses.^[^ ^35^, ^36^ ^]^ Therefore, if DNA repair response pathways were up‐regulated in TP‐T_M_^B16^ versus TP‐T_M_^MC38^, then accordingly, TP‐T_M_^B16^ may be more senescent than TP‐T_M_^MC38^. Based on the guidelines for experimental validation of cellular senescence,^[^ ^37^, ^38^ ^]^ we first analyzed the transcriptional markers of cellular senescence in the gene expression data. Since cellular senescence phenotypes are highly heterogeneous across different organs and cell types,^[^ ^38^ ^]^ we opted to compare transcriptional signatures using gene lists from the Reactome database that were used to define cellular senescence (Table S1, Supporting Information). Accordingly, we performed GSEA comparing TP‐T_M_^B16^ or TP‐T_M_^MC38^ to the more conventional VP‐T_M_ (Figure 6b). Gene sets that defined “cellular senescence”, “senescence‐associated secretory phenotype” (SASP), and “DNA damage/telomere stress induced senescence” showed positive enrichment for both TP‐T_M_^B16^ and TP‐T_M_^MC38^ compared to VP‐T_M_, suggesting that TP‐T_M_^B16^, and surprisingly TP‐T_M_^MC38^, were senescent. However, TP‐T_M_^B16^ showed a much higher normalized enrichment score relative to TP‐T_M_^MC38^. In addition, gene sets that defined “oxidative stress induced senescence” only showed positive enrichment for TP‐T_M_^B16^ and not TP‐T_M_^MC38^. Overall, TP‐T_M_^B16^ showed greater cellular senescence and senescence sub‐type enrichment than TP‐T_M_^MC38,^ signifying a marked difference in overall senescence severity.

To further validate these findings, we isolated TP‐T_M_^B16^, TP‐T_M_^MC38^, and VP‐T_M_ (Figure 6c), and stained them for the detection of lipofuscin, an aggregate of heavily oxidized proteins/lipoproteins and metals that accumulate due to senescence‐associated oxidative damage. We observed a statistically significant increase in lipofuscin detection when comparing TP‐T_M_^B16^ to VP‐T_M,_ but an insignificant change when comparing TP‐T_M_^MC38^ to VP‐T_M_ (Figure 6d)_._ This is in contrast to our detection of senescence‐associated β‐galactosidase (SA‐β‐gal), where we observed a statistically significant increase in SA‐β‐gal in both TP‐T_M_^B16^ and TP‐T_M_^MC38^ compared to VP‐T_M_ (Figure 6e). Finally, as previously mentioned in Figure 3, when comparing TP‐T_M_^B16^ to VP‐T_M_, *CD27* was down‐regulated 7.1‐fold while *SPARC* was up‐regulated 304‐fold. CD27 down‐regulation and SPARC up‐regulation are known senescence markers^[^ ^39^, ^40^, ^41^ ^]^ and showed the greatest differential expression amongst analyzed senescence‐related genes. Flow cytometric staining of CD27 showed significant down‐regulation when comparing TP‐T_M_^B16^ to VP‐T_M,_ but an insignificant change when comparing TP‐T_M_^MC38^ to VP‐T_M_ (Figure 6f). In contrast, we observed a statistically significant increase in SPARC in both TP‐T_M_^B16^ and TP‐T_M_^MC38^ compared to VP‐T_M_, and more importantly, a significant increase when comparing TP‐T_M_^B16^ to TP‐T_M_^MC38^ (Figure 6g). Ultimately, TP‐T_M_^MC38^ may express some transcriptional and phenotypic indicators of cellular senescence, but the increased senescence severity observed in TP‐T_M_^B16^ may explain its predisposition for p21‐dependent cell cycle arrest and poor antitumor capacity.

## 3 Discussion

The unreliable clinical success of T cell therapies in the treatment of solid tumors can be attributed to the heterogeneity of T cell dysfunctional states that are induced during tumor growth. In this paper, we reported that IFNAR blockade restored the therapeutic response of endogenous tumor‐specific memory T cells that displayed the features of cellular senescence. In the presence of IFN‐I, these cells had limited proliferative capacity during boosting oncolytic viral vaccination. This was associated with DNA damage response/cell cycle arrest‐related pathway enrichment and relied on p21/CDKN1A signaling since p21 inhibition enhanced T cell responses and tumor regression. While many tumor lines such as B16‐gp33, LLC, MCA205, MO5, and B16F10 required IFNAR blockade for therapeutic efficacy, MC38‐gp33 did not. Endogenous memory T cells derived from MC38‐gp33 showed reduced cellular senescence compared to B16‐gp33, suggesting that hypersensitivity to IFN‐I is a pre‐encoded feature of cells that expressed greater senescence severity. This work associates IFN‐I signaling with dysfunctional T cell outcomes, and thus has important implications in the rational design of cancer vaccines as they are often formulated to maximize IFN‐I induction.

Oncolytic rhabdoviruses, including VSV and Maraba, have been shown to be able to selectively replicate in and kill tumor cells with minimal impact on normal tissue.^[^ ^42^, ^43^ ^]^ When engineered to express tumor antigens, they are also suitable for stimulating potent CD8+ responses from tumor antigen‐experienced T cells and promoting their recruitment into the TME via local inflammatory remodeling.^[^ ^44^, ^45^ ^]^ Using a heterologous viral vaccine or ACT to create a tumor antigen‐experienced T cell reservoir, we have demonstrated extensively that OVV can potently regress established tumors.^[^ ^32^, ^46^ ^]^ While these viruses have mechanisms to evade cellular antiviral responses, VSVΔM51 which was used in this study, has a deletion that allows for late gene transcription of type I interferon‐related genes as an intentional design choice to enhance the safety of the virus and tumor tropism.^[^ ^47^, ^48^ ^]^ As such, virus‐infected cells can become a source of exogenous IFN‐I that can be sensed by endogenous tumor‐infiltrating T cells. Here, we confirm that IFN‐I is not only dispensable for OVV‐mediated secondary T cell expansion, but actively contributes to treatment failure by promoting dysfunctional outcomes from senescent tumor‐primed memory T cells.

In cancer, senescent T cells are uniquely defined by a persistent DNA damage response (DDR) due to genotoxic stress from the TME. While cell cycle arrest is not unique to senescent T cells, DNA damage can perpetuate cell cycle arrest to maintain poor proliferative responsiveness even during antigen stimulation.^[^ ^49^ ^]^ It is possible that IFN‐I hyperresponsiveness converges upon these processes through the cGAS‐STING pathway, which has been highly associated with cellular senescence.^[^ ^50^, ^51^, ^52^ ^]^ Activation through DNA damage sensing can lead to IRF3/NFκB activation and promote a pro‐inflammatory response which includes intrinsic IFN‐I production.^[^ ^53^, ^54^, ^55^ ^]^ In turn, extracellular sensing of intrinsically produced IFN‐I (as well as extrinsic sources of IFN‐I) may amplify DDRs in response to DNA damage.^[^ ^56^, ^57^, ^58^ ^]^ Indeed, HIN‐200 family proteins (IFN‐I‐inducible cytosolic DNA sensors), specifically IFI16, have been shown to increase dramatically through IFNβ, and their expression have been associated with permanent cell cycle arrest and cellular senescence.^[^ ^59^, ^60^, ^61^ ^]^

T cell senescence is a distinct dysfunctional state compared to T cell exhaustion. While the induction of T cell senescence during cancer is associated with various microenvironmental stressors that promote DNA damage, T cell exhaustion is associated with continuous or poor antigen stimulation. However, despite unique developmental and regulatory programs, both dysfunctional states impair effector function, reduce proliferative activity, and possess overlapping characteristics.^[^ ^49^ ^]^ Indeed, while we observed that TP‐T_M_^B16^ down‐regulated typical markers of exhaustion such as Tim3, CD38, and CD39, we were surprised to find that PD‐1 was up‐regulated. However, while PD‐1 is conventionally viewed as a marker of exhaustion, it has also been associated with cellular senescence.^[^ ^62^ ^]^ Regardless, senescent T cells are distinct in terms of their molecular signaling and in their secretory phenotype,^[^ ^49^, ^63^ ^]^ which we have validated here.

Interestingly, TP‐T_M_^B16^ and TP‐T_M_^MC38^ seem to diverge from classical definitions of senescence in ways that can have important therapeutic implications. While it has been traditionally accepted that senescence is associated with irreversible proliferative arrest,^[^ ^64^, ^65^ ^]^TP‐T_M_^MC38^ challenge this notion in that TP‐T_M_^MC38^ do not seem to suffer proliferative arrest. It is possible that, similar to exhausted T cells, senescent T cells can exist on a phenotypic continuum where increasing acquisition of senescence features correlates with increasing dysfunction. IFN‐I hyperresponsiveness might thus represent a unique senescence feature that is found further along the continuum. In the literature, it has been reported that the transcriptome of senescent cells is highly heterogenous and temporally dynamic;^[^ ^66^, ^67^ ^]^ similarly, intermediate senescence phenotypes have also been reported.^[^ ^68^, ^69^ ^]^ Therefore, TP‐T_M_^MC38^ may represent an intermediate senescence phenotype that can respond to OVV treatment and is inert to IFN‐I signaling‐mediated dysfunction.

TP‐T_M_^B16^ also challenge the idea of irreversible proliferative arrest since their secondary expansion during VSV‐gp33 treatment could be rescued with IFNAR blockade. To reconcile this, it was recently acknowledged that cell‐cycle arrest‐related dysfunction in exhausted T cells can be reversed by immune checkpoint blockade.^[^ ^70^ ^]^ Rather than reinvigorating TCF1‐ PD1+ terminally exhausted effector T cells, the therapeutic efficacy of ICB was primarily attributed to the expansion of TCF1+ PD1+ precursor exhausted memory T cells.^[^ ^17^, ^71^ ^]^ As a result, memory differentiation may dictate whether exhausted T cell reservoirs can be rescued to promote tumor rejection. Interestingly, classical definitions of cellular senescence suggest that cell‐cycle arrest‐related dysfunction cannot be reversed,^[^ ^72^, ^73^ ^]^ and that it is a reflection or consequence of terminal differentiation.^[^ ^65^, ^74^, ^75^ ^]^ However, there are also reports that cellular senescence can indeed be reversed,^[^ ^76^, ^77^, ^78^ ^]^ and in our own hands, we observed that the proliferation and therapeutic efficacy of TP‐T_M_^B16^ can be rescued within the context of IFNAR blockade. The important distinction in our case is that we reported cellular senescence in an endogenous tumor‐specific memory T cell pool. Taken together, it is possible that, similar to exhausted T cells, cellular senescence can be expressed in both memory and effector T cells, and that an effective approach to reverse cellular dysfunction would be to adopt a strategy that reinvigorates “precursor senescent memory T cells”.

Ultimately, endogenous tumor reactive T cell reservoirs have the capacity for rejecting solid tumors. However, they are regularly suppressed by a heterogeneity of factors that can promote various forms of T cell dysfunction, such as senescence. As a result, immunotherapeutic approaches that depend on the expansion of these cells such as oncolytic viral vaccination, etc. display limited efficacy in the treatment of solid tumors. If IFN‐I hyperresponsiveness is an advanced feature of T cell senescence, quantification of senescence severity in human solid cancers may rationalize the use of IFN‐I‐blocking therapeutics for these patients. As a result, the dysfunction caused by cellular senescence may be alleviated to rescue tumor‐primed memory T cell proliferation and restore immunotherapeutic efficacy.

## 4 Experimental Section

### Study Design

The objective of this study was to investigate how IFNAR blockade during oncolytic viral vaccination reinvigorates endogenous senescent‐like tumor‐specific memory T cells. In vivo experiments were conducted to assess the differential therapeutic effects of IFNAR blocking antibody and to characterize endogenous senescent‐like tumor‐specific memory T cells before and after treatment.

### Animal Models

Age‐matched (6–8 weeks old) mice were used for all experiments. For in vivo studies, group sizes ranged from *n* = 4–5, while ex vivo analyses used *n* = 3–5 per group. Experiments were replicated at least twice to ensure reproducibility. Tumor‐challenged mice were randomized prior to treatment, and all treatments were administered in a blinded manner to minimize bias. Mice were housed in a pathogen‐free environment at the Central Animal Facility at McMaster University. C57BL/6 mice were purchased from Charles River Laboratories. Thy1.1 mice (B6.PL‐Thy1a/CyJ) were purchased from The Jackson Laboratory. P14 mice (B6.Cg‐Tcratm1Mom Tg(TcrLCMV)327Sdz), a transgenic mouse strain that carries a TCR recognizing an H‐2Db–restricted epitope of LCMV‐GP33–41 (KAVYNFATM), were purchased from Taconic Breeding Laboratories and were cross‐bred to Thy1.1 mice to generate P14‐Thy1.1 mice.

### Sex as a Biological Variable

This study primarily examined female mice, but used male mice in repeats of therapy‐related studies. Similar findings were observed for both sexes. Accordingly, phenotypic characterization of T cells was primarily done in female mice. It was unknown whether the same phenotype could be observed in male mice.

### Animal Study Approval

Mice were monitored daily for signs of distress. Humane endpoints were defined by decreased body condition score or tumor volume exceeding 1000 mm^3^. Veterinary staff conducted daily welfare checks and notified researchers when humane endpoints were reached. All animal experiments were compliant with Canadian Council on Animal Care guidelines and received internal approval through the McMaster University Animal Research Ethics Board. The animal ethics approval number was AUP‐23‐46.

### Tumor Cell Culture

B16F10, B16‐gp33, MO5 (B16‐OVA), MC38‐gp33, MCA205‐gp33, and LLC‐gp33 were maintained at 37 °C in a humidified atmosphere with 5% CO_2_. gp33‐expressing cell lines were engineered by lentiviral transduction to express a minigene corresponding to the LCMV gp33‐41 peptide (M‐KAVYNFATM).^[^ ^79^ ^]^ Cells were cultured in MEM/F11 containing 10% FBS, 2 mm l‐glutamine, 5 ml sodium pyruvate, 5 mL nonessential amino acids, 5 mL vitamin solution, 55 µm 2‐mercaptoethanol, 100 U mL^−1^ penicillin, and 100 ng ml^−1^ streptomycin.

### Viruses

VSV‐gp33 is a recombinant vesicular stomatitis virus expressing the expressing the LCMV gp33‐41 peptide (M‐KAVYNFATM) minigene.^[^ ^80^ ^]^ VSV‐DCT and VSV‐SIINFEKL were previously described.^[^ ^14^ ^]^ All VSV vectors carry a deletion of the methionine residue at position 51 of the matrix protein (ΔM51) as described previously.^[^ ^81^ ^]^ LCMV‐Armstrong was utilized for acute infection of mice to generate a antigen‐specific CD8+ T cell response against gp33.^[^ ^82^ ^]^

### Peptides

Peptides for gp33 (KAVYNFATM), OVA (SIINFEKL), and DCT (SVYDFFVWL), were purchased from Biomer Technologies and dissolved in PBS supplemented with 0.5% BSA.

### T_CM_ Culture

The culture method for generating memory T cells for ACT was previously described.^[^ ^19^ ^]^ Briefly, bulk splenocytes derived from gp33‐specific TCR‐transgenic P14‐Thy1.1 mice were stimulated with gp33 peptide (100 ng mL^−1^) in the presence of 10 ng mL^−1^ IL15, IL21, and 20 ng mL^−1^ rapamycin for seven days in complete RPMI (10% FBS, 2 mm l‐glutamine, 55 µm 2‐mercaptoethanol, 100 U mL^−1^ penicillin, and 100 ng ml^−1^ streptomycin) prior to injection.

### Tumor Challenge

For implantation, trypsinized tumor cells were washed twice with PBS and resuspended in PBS at a concentration of 10^6^ cells/30 µL for LLC‐gp33 cells or 10^5^ cells/30 µL for B16‐gp33, MO5, MCA205‐gp33, and MC38‐gp33 cells. Mice were challenged via intradermal injection, and tumors were allowed to grow to a mean volume of ≈150 mm^3^ prior to the commencement of treatment (≈ 7 days).

### TP‐T_M_ and VP‐T_M_ Isolation

TP‐T_M_ were generated by intradermal tumor challenge and allowing tumors to grow to a mean volume of ≈150 mm^3^, ≈7 days. VP‐T_M_ were generated by infecting mice acutely with LCMV‐Armstrong at a concentration of 2 × 10^5^ pfu and waiting for 7–30 days. In any experiment that involved the co‐analysis of TP‐T_M_ and VP‐T_M,_ the memory T cell generation time was normalized to seven days to mitigate age‐related influences on cellular senescence induction. Inguinal lymph nodes, and/or spleens were isolated and mechanically dissociated in complete RPMI. Cells were sorted or analyzed based on CD8, gp33 tetramer (gp33H2DbTet), and T cell differentiation marker (CD44, CD62L, CD127, and KLRG1) staining.

### Tumor Treatment

Mice were injected intravenously with 2 × 10^8^ pfu of VSV‐gp33 in 200 µL PBS. αIFNAR (1 mg, one dose; clone MAR1‐5A3; InVivoMab) antibodies were administered by intraperitoneal injection. P21 inhibitor UC2288 was purchased from MedChemExpress, dissolved in DMSO, and injected intraperitoneally at (10 mg kg^−1^ body weight) in 100 µL PBS.

### Surface and Intracellular Staining of T Cells

gp33 tetramer (gp33H2DbTet) was purchased from Tetramer Baylor College MHC Tetramer Production Laboratory. Fluorophore‐conjugated SPARC (124 413) antibody was purchased from R&D Systems. Fluorophore‐conjugated p21 (EPR18021) antibody was purchased from Abcam. The following fluorophore‐conjugated antibodies were purchased from BD Biosciences: CD8a (53‐6.7), Thy1.1 (OX‐7), IFN‐γ (XMG1.2), CD62L (MEL‐14) CD44 (IM‐7), CD127 (SB/199), KLRG1 (2F1), Ki‐67 (B56), CD27 (LG.3A10). Blood and tumor‐draining lymph node samples were collected and treated with ACK lysis buffer (Thermo Fisher Scientific) to remove red blood cells prior to peptide stimulation and/or staining. Surface staining, fixation/permeabilization (Cytofix/Cytoperm, BD Biosciences), and intracellular staining were previously described.^[^ ^19^ ^]^ For analysis of antigen‐specific responses, PBMCs were stimulated with peptide (1 µg mL^−1^) in culture at 37 °C for 4 h. Brefeldin A (GolgiPlug, BD Biosciences; 1 µg mL^−1^) was added for the last 3 h of incubation. For staining lipofuscin, biotinylated SBB‐Analogue (GL13) (MedChemExpress) was incubated with isolated cells according to manufacturer instructions and then stained with fluorophore‐conjugated streptavidin (Biolegend). For staining SA‐β‐gal, CellEvent Senescence Green Flow Cytometry Assay Kit (Thermo Fisher Scientific) was used according to manufacturer instructions. CFSE staining was conducted as described.^[^ ^83^ ^]^

### Transcriptome Microarray Analysis

gp33H2DbTet^+^ CD8^+^ T cells were isolated, stained and flow sorted (FACSAria III, BD Biosciences) from tumor‐draining lymph nodes. RNA was extracted (RNeasy Mini, QIAGEN) and prepared for gene expression analysis using a Clariom S array (Applied Biosystems). Sample preparation for microarray hybridization was conducted with a GeneChip WT Pico Reagent Kit (Applied Biosystems), and samples were run on a GeneChip Scanner 3000 (Applied Biosystems). CEL files were analyzed using Transcriptome Analysis Console (TAC 4.0), and probe set signals were SST‐RMA‐normalized prior to further analysis.

### Gene Ontology Enrichment Analysis

Genes were considered differentially expressed if the fold change was >2.0 and *q* value was <0.1. Enrichment analysis was conducted by PANTHER overrepresentation test using the Gene Ontology (GO) database with GO biological process complete annotation dataset.^[^ ^84^, ^85^, ^86^ ^]^ The reference gene list was the *Mus musculus* genome, and expression data analyses includes Bonferroni correction for multiple testing. Overrepresented GO terms were sorted hierarchically and displayed results were statistically significant (*p* < 0.05).

### Gene Set Enrichment Analysis

Utilizing GSEA4.1,^[^ ^87^ ^]^ enrichment analysis was conducted using Hallmark and C2 gene sets from the Molecular Signatures Database (MSigDB).^[^ ^87^ ^]^ Network creation was done using the EnrichmentMap plugin^[^ ^88^ ^]^ and AutoAnnotate plugin^[^ ^89^ ^]^ for Cytoscape.^[^ ^90^ ^]^ The following parameters were used: number of permutations, 2000; permutation type, gene set; metric for ranking genes, t‐test. The ssGSEA (v10.0.x) projection module^[^ ^91^ ^]^ within GenePattern^[^ ^92^ ^]^ was used to calculate separate enrichment scores for individual sample‐gene set pairings, independent of phenotype labeling. ssGSEA was performed on untransformed gene expression signal values using the following parameters: sample normalization method, rank; weighting component, 0.75; minimum gene set size, 10. Pearson's correlation coefficient was used to calculate the correlation between enriched Hallmark pathways (ssGSEA enrichment scores) and visualized as a correlation matrix or lollipop plots.

### Statistics

GraphPad Prism was used for graphing and statistical analyses. Immune response data were analyzed with unpaired Student's t‐test or one‐way ANOVA. Where necessary, Tukey's test was used to correct for multiple comparisons. Overall, a p‐value of less than 0.05 was considered significant. Mean + SEM bars were shown. Survival curves were generated using the Kaplan‐Meier method and analyzed using the log‐rank (Mantel‐Cox) test.

## Conflict of Interest

The authors declare no conflict of interest.

## Author Contributions

A.N., S.R.W., L.D., and L.C. performed the experiments, analyzed the data, and assisted in manuscript preparation. A.N., S.R.W., and Y.W. oversaw experimental design and data interpretation and prepared the manuscript.

## Supporting information



Supporting Information

Supporting Information

## Data Availability

The data that support the findings of this study are openly available in Gene Expression Omnibus at https://www.ncbi.nlm.nih.gov/geo/query/acc.cgi?acc=GSE305024, reference number 305024.
