# Supplementary material for: Pre‐Encoded IFN‐I Sensitivity Exacerbates Memory T Cell Senescence in Solid Tumors
Source: Adv Sci (Weinh). 2025 Oct 5;13(35):e04474. doi: 10.1002/advs.202504474 (PMC13292250; doi:10.1002/advs.202504474)
Supplement: Supplementary file 1 — Supporting Information [file ADVS-13-e04474-s001.pdf]

Supplementary Data

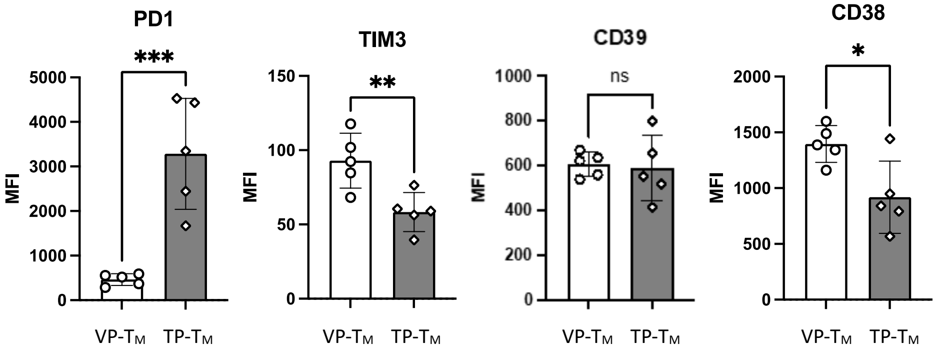

Supplementary Figure 1: Expression of exhaustion markers on TP-T_M compared to VP-T_M.

C57BL/6 mice were challenged i.d. with B16-gp33 tumors in order to generate TP-T_M or acutely infected i.v. with LCMV in order to generate VP-T_M. Memory T cells were isolated from secondary lymphoid tissue and identified based on CD8, gp33H2DbTet, and memory differentiation marker (CD44, CD62L, CD127, KLRG1) expression before staining for exhaustion markers.

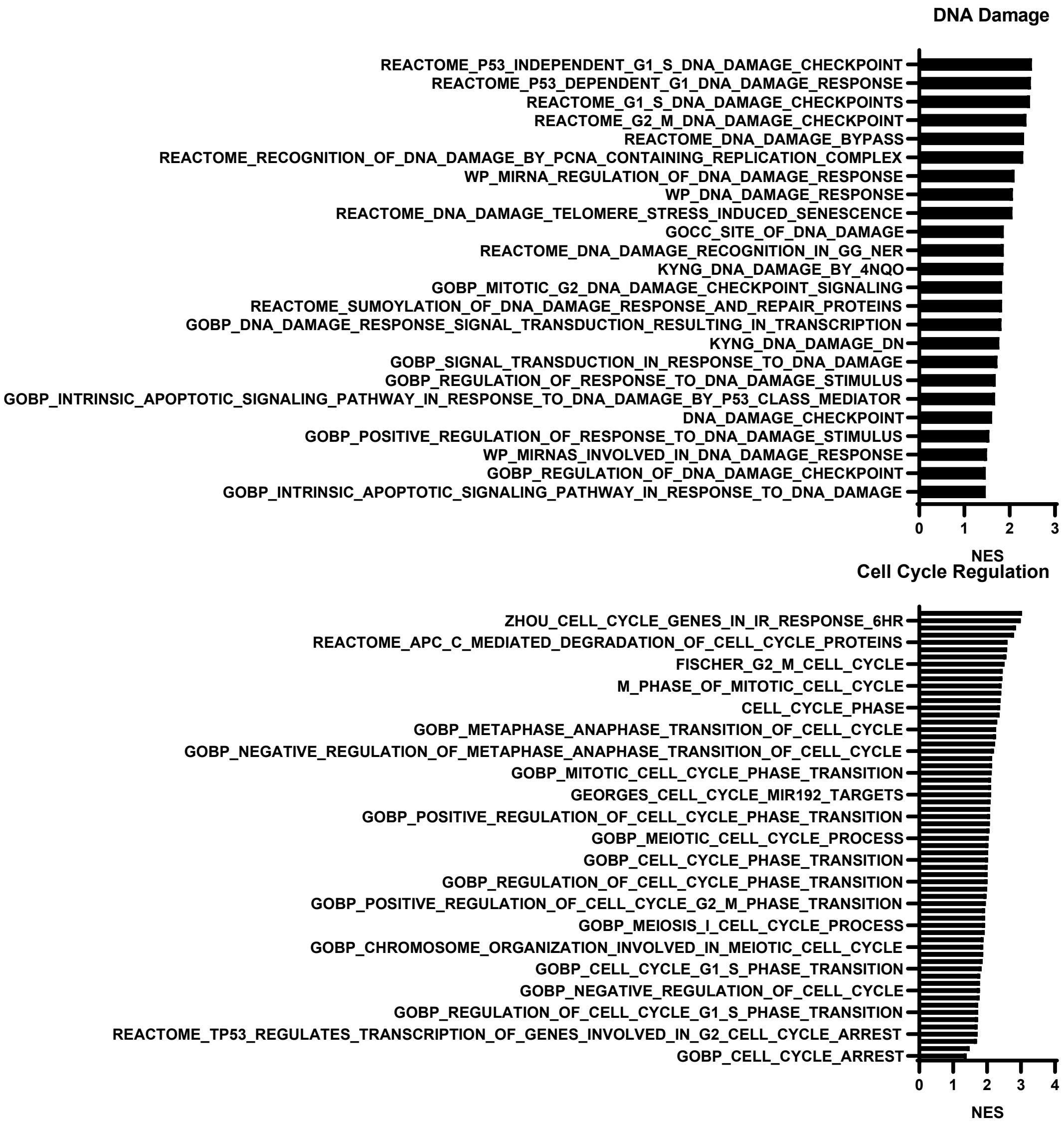

Supplementary Figure 2: Gene set enrichment of DNA damage and cell cycle-related pathways. TP-T_M and VP-T_M were compared by GSEA using MSigDB C2 manually curated pathways that were manually screened for cell cycle arrest-related and DNA damage-related pathways.

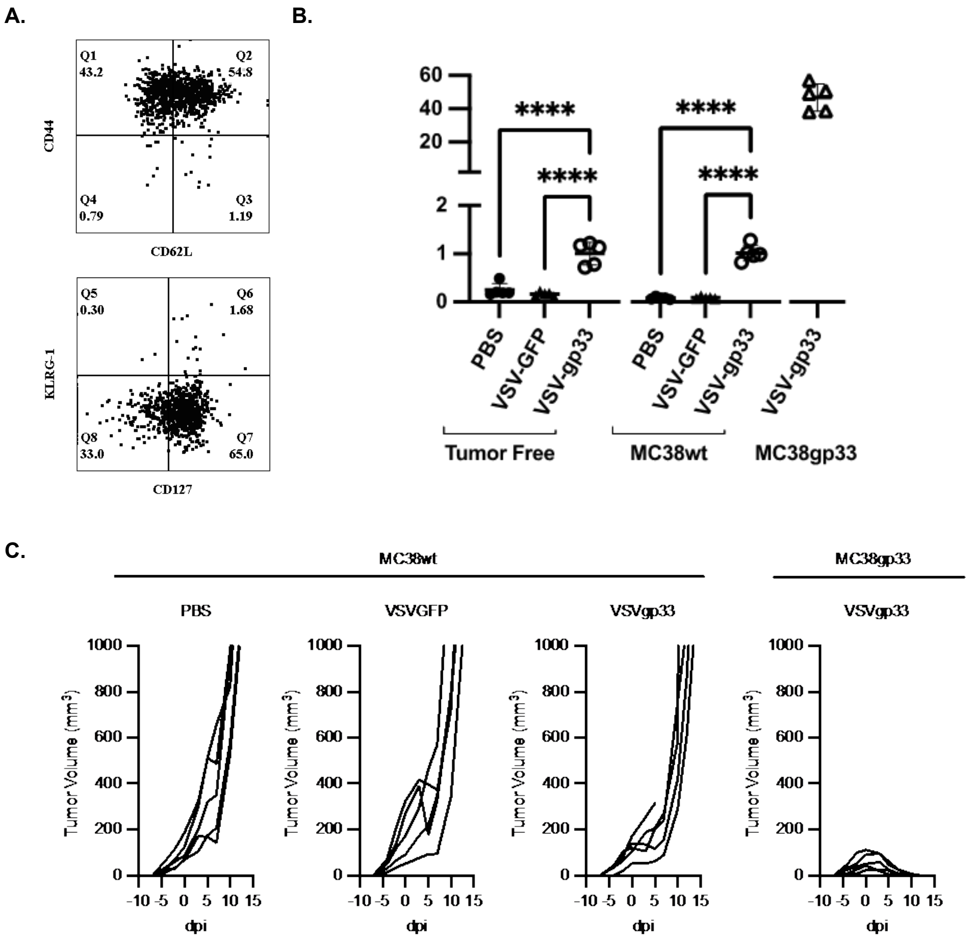

Supplementary Figure 3: Tumor primed T cells dominate response to VSV vaccination

(A) Phenotype of gp33-specific T cells in the tumor draining lymph nodes of MC38gp33 tumor bearing mice. (B) Quantification of gp33-specific T cell responses in the circulation mice bearing the indicated tumor five days after treatment with correlated tumor volumes (C).
